# Urban-Rural Differences in Nutritional Status and Dietary Intakes of School-Aged Children in Cambodia

**DOI:** 10.3390/nu11010014

**Published:** 2018-12-20

**Authors:** Yoko Horiuchi, Kaoru Kusama, Sar Kanha, Nobuo Yoshiike, the FIDR Research Team

**Affiliations:** 1 Graduate School of Health Sciences, Aomori University of Health and Welfare, 58-1 Mase, Hamadate, Aomori 030-8505, Japan; 1693001@ms.auhw.ac.jp (Y.H.); n_yoshiike@auhw.ac.jp (N.Y.); 2 Department of Food and Health Sciences, Faculty of Health and Human Development, The University of Nagano, 8-49-7 Miwa, Nagano 380-8525, Japan; 3 Strengthening Public Health Laboratory, Workforce Development, and Health Systems Research Capacity of the Cambodian National Institute of Public Health, Lot #80, St. Samdach Penn Nouth Blvd (289), Boeung Kok 2 Commnue, Toul Kork District, Phnom Penh 12152, Cambodia; sar.kanha.lucie@gmail.com

**Keywords:** Southeast Asia, Cambodia, urban-rural, school-aged children, nutritional status, dietary intake

## Abstract

This study aimed to describe the nutritional status and dietary patterns of Cambodian school-aged children compared with those in the South East Asian Nutrition Survey (SEANUTS; Indonesia, Malaysia, Thailand, and Vietnam in 2011) and to clarify the urban-rural differences using data from a nationally representative sample. The survey was conducted in 2014/2015 with a sample of 2020 children aged 6–17 years from 136 randomly selected schools. Standardized anthropometric measurements and a 1-day dietary survey by 24-h recall method were conducted. Extended analyses in the present study revealed that the difference between rural and urban areas was similar to that of the SEANUTS; the overall prevalence of stunting remained high (33.2%). Stunting was more prevalent in children living in rural areas than in those in urban areas (total: 36.4% vs. 20.4%). In contrast, the overall prevalence of overweight and obesity was not as high (3.1%), but was higher among urban children in all age groups compared with those living in rural areas (total: 6.4% vs. 2.3%). Overall, the dietary intake of children did not meet the local recommended dietary allowances, which was similar to the results of the SEANUTS and differed across urban and rural areas.

## 1. Introduction

Cambodia is a Southeast Asian country, and the proportion of children aged 6–17 years was 24.6% of the total population (14,677 million, 2013) [1]. Nutritional status in early childhood may greatly affect health, and subsequently impact not only a child’s own growth and development, but also economic development in the country [2,3]. Several surveys targeting children under 5 years of age have been conducted in the country, and nutrition polices for this segment have been implemented [4]. However, nutrition surveys or policies for school-aged children are limited [5,6]. Nutritional conditions, not only under 5 years, but also during the school-aged period, affect cognitive ability and subsequently influence the next generation [6,7,8,9]. Four countries in Southeast Asia (Thailand, Vietnam, Indonesia, and Malaysia) have conducted nationwide surveys in response to the need for information on the nutritional status of young children, as the South East Asian Nutrition Survey (SEANUTS) [10,11,12,13,14,15]. In the survey, anthropometric measurements and dietary assessment for children aged 0.5–12 years were obtained to provide insight on the nutritional status of children in 2013 [10,11,12,13,14,15]. Results of this study showed that stunting was a major nutritional problem in Indonesia (rural: 39.2%; urban: 25.1%) compared with Malaysia (rural: 8.8%; urban: 8.3%), Thailand (rural: 8.4%; urban: 4.1%), and Vietnam (rural: 17.5%; urban, 7.9%). In Indonesia, Thailand, and Vietnam, stunting and thinness were more common in rural areas. Overweight and obesity were higher among urban children in all countries (Vietnam, 29.0%; Malaysia, 22.4%; Thailand, 18.7%; and Indonesia, 10.7%) compared with those in rural areas (Vietnam, 5.6%; Malaysia, 18.1%; Thailand, 13.9%; and Indonesia, 5.0%) [11,12,13,14,15]. Overall, the dietary intake of children did not meet the recommended dietary allowances [11,12,13,14], and differed between urban and rural areas [11,14]. These preliminary results from the SEANUTS revealed that there are regional differences in the double burden of malnutrition. In Cambodia, surveys targeting children aged 6–16 years in 2012 [5] and 13–15 years in 2007–2014 [16] were conducted. However, sampling of these surveys was performed in limited places and urban-rural differences were not mentioned. To rectify the lack of information for the nutritional status of school-aged children in Cambodia, the Foundation for International Development/Relief (FIDR) started a project that included a nationwide nutritional survey of the country’s children in collaboration with the Department of Preventive Medicine of the Cambodian Ministry of Health, with technical advice provided by the authors in the present study between November 2014 and July 2015, with a sample that included 2020 children aged 6–17 years old. Although the basic and descriptive results were published as a survey report [17], no analytic demonstrations, including urban-rural differences, were shown. The authors performed extended analyses for the present study in collaboration with the FIDR to describe the nutritional status and dietary patterns of Cambodian school-aged children compared with four countries in Southeast Asia and to clarify the urban-rural differences using the data from the nationally representative sample.

## 2. Materials and Methods

The survey was administered to a nationwide representative sample from 23 provinces and Phnom Penh at 136 schools from November 2014 to July 2015 by the FIDR, with members from relevant departments of the Royal Government of Cambodia, international organizations, and non-governmental organizations. A multistage cluster sampling stratified by geographical location, sex, and age was carried out to recruit children aged 6–17 years by using Emergency Nutrition Assessment for Standardized Monitoring and Assessment of Relief and Transitions (ENA for SMART) 2011 (Toronto, Canada) [18], and a total of 2020 samples (959 boys and 1,061 girls) were collected. Ethical approval was obtained on August 25, 2014 from the National Ethics Committee for Health Research of the Ministry of Health of Cambodia.

### 2.1. Anthropometric Measurements and 24-h Dietary Recall

Weight and height of children were measured according to an anthropometric protocol based on the World Health Organization standard [19]. All measures were taken twice and the mean value was used for analysis. At the same time, a 24-h dietary recall survey was conducted by trained interviewers to record foods and drinks that the respondents consumed throughout that day. First, the respondents were asked whether the previous day was a usual one, that is, household foods were consumed, not any special foods that are served during ceremonies, parties, and so on; the data were deleted in cases of an unusual day. Next, a dietary record form was used to record the foods and drinks that the respondents consumed, and photos or pictures were used to assist the quantification [17]. The individual amount consumed was considered as a priority, although the amount of household food consumed was included to cross check information during the interview.

### 2.2. Statistical Analyses

The anthropometric status of the respondents was determined based on the WHO growth reference for those aged 5–19 years. Height-for-age z-score (HAZ) and Body mass index-for-age z-score (BAZ) were determined using the Antho Plus software (version 3.2.2, Geneva, Switzerland) for children aged >5 years. The cut-off value for underweight, stunting, and thinness was −2 standard deviation (SD). For overweight and obesity, the cut-off value of 1 and 2 SD was used for children aged >5 years [20].

Nutrient analysis was performed using the FIDR Nutrition Calculation Database 2013, which was developed based on the Association of Southeast Asian Nations (ASEAN) Food Composition Tables [21] and Sustainable Micronutrient Interventions to Control Deficiencies and Improve Nutritional Status and General Health in Asia project (SMILING) food composition table for Cambodia [22]. Statistical analysis was performed using IBM SPSS statistics (version24, Chicago, IL, USA). All analyses were performed on weighted data. Weight factors were calculated to extrapolate study participants to the national estimate [11,12,13,14]. The weighting method was based on the actual number of children by age, sex, and area of residence using the population reports [1]. Descriptive statistics are presented as weighted population means with standard errors or percentages for prevalence. The children were clustered into four groups, namely, 6–9, 10–12, 13–15, and 16–17 years. Differences between variables in different strata (age group, area of residence, and sex) were tested for significance using analysis of covariance, after adjusting for age. Furthermore, differences in the proportion of under and overnutrition were examined using an χ^2^ test. A two-sided *p* value of *p* <0.01 or 0.05 was considered significant.

## 3. Results

Table 1 presents the number of children in the four age strata by sex and area of residence as well as the estimated population of children in Cambodia. A total of 2020 children participated in the study, representing an estimated population of 3,609,517 children aged 6–17 years in Cambodia. Approximately 47.5% (959 of 2020) of the recruited children were boys and 19.9% (402 of 2020) resided in urban areas, which was almost similar to that of the population.

Table 2 reports the means and standard errors for anthropometric and characteristics by age group and residential area. Children in urban areas tended to have greater weight and height than those in rural areas. In particular, among the group aged 6–9 years, all anthropometric characteristics showed significant differences between areas of residence (*p* < 0.01). The HAZ and BAZ values were all negative irrespective of age group, sex, and area of residence. Stunting and thinness were more prevalent in rural children than in their urban counterparts. Overall, the prevalence of stunting among rural children was 36.4% and that among urban children was 20.4%. The prevalence of overweight and obesity among rural children was 2.3% and that among urban children was 6.4%. The highest prevalence of stunting (50.8%) was observed among rural boys aged 13–15 years.

Table 3 shows the means and standard errors for intakes of selected nutrients, and the proportion of energy from macronutrients. Urban children generally had a higher intake of protein and fat than their rural counterparts. Overall, the proportion of energy from carbohydrate was higher in rural children than in their urban counterparts; in contrast, the proportion of energy from protein and fats were lower in rural children than in their urban counterparts, with significant differences.

Table 4 shows the percentage of children in each age group with energy and nutrient intake below the local dietary requirement (CAM-RDA) [17] stratified by sex and area of residence. Higher percentages were observed in rural areas than in urban areas, and 73.1% of children did not meet the CAM-RDA for energy, 86.1% for calcium, and 44.9% for iron.

Table 5 reports the means and standard errors for dietary intakes by food groups. We included the reported intake of food groups; however, this was not reported in the results of the SEANUTS. Rural children had a higher intake of staple foods than urban children. In contrast, urban children had higher intakes of meats and eggs than rural children among all age groups and sexes, with a significant difference; moreover, dairy products and fats/oils tended to be higher in urban children than in rural children among all age groups and sexes.

## 4. Discussion

### 4.1. Anthropometric Condition

The results show that the HAZ and BAZ of Cambodian school-aged children were both below the WHO standards [20] for all age groups and sexes and that undernutrition remains a problem in Cambodia. The negative value of HAZ was associated with a high percentage of stunting. Similar to the results of the SEANUTS, the prevalence of stunting and thinness was higher in rural children than in urban children, regardless of sex and age group [11,12,13,14,15]. Children in urban areas had better nutritional status than those in rural areas, and there is a nutritional disparity between urban and rural areas [11]. The overall prevalence of stunting in this study (33.2%) was lower than the results of the survey among Cambodian children aged 6–16 years old conducted in 2012 (40.0%) [5]. Regarding the growth of children under 5 years in Cambodia, the percentage of children with stunting fell consistently [23]. This finding might be related to the improvement in the growth of school-aged children in Cambodia. Rural children had a higher percentage of stunting than urban children, especially boys aged 13–15 years living in rural areas (50.8%). Boys living in rural areas in Indonesia also had a higher prevalence of stunting [11].

This result might be due to the fact that stunting has always been related to wealth, as sources of protein are relatively expensive, and ensuring dietary diversity is difficult for poor families. In addition, growth velocity in boys is higher; thus, their nutrient requirements are higher [11]. According to a review study conducted in 2007–2013 (13–15 years) [16], overweight and obesity was 3.7%. In this study, the prevalence of overweight and obesity among the same age group was 3.1%. Overall, overweight and obesity was found to be more prevalent in urban areas than in rural areas, which is similar to that reported in the SEANUTS [11,12,13,14]. The percentage of overweight and obesity was not so high compared with that in the four countries included in the SEANUTS.

### 4.2. Nutritional Intake Situation

Urban children had a higher prevalence of protein and fat intake than rural children; this finding is similar to that of school-aged children included in the SEANUTS. Rural children had a higher percentage of energy from carbohydrates, but a lower percentage of energy from proteins/fats; this finding is similar to that observed in Vietnam [14] as reported in the SEANUTS. This might be related to the lower intake of fat- and oil-rich food and the higher intake of staple foods among rural children. Higher intakes of meats/eggs and dairy products were observed in urban children than in their rural counterparts. Consumption of animal source foods was found to be associated with a decreased risk of stunting; the higher prevalence of stunting among rural children might be due to the lower consumption of animal source foods [24]. A study in Malaysia reported that the percentage of children who did not achieve the Malaysian Recommended Nutrition Intakes (RNI) for calcium was higher among school-aged children than among infants and toddlers. Milk, a food source rich in calcium, is the dominant food for infants and toddlers [12]. This might explain the higher proportion of school-aged children (86.1%) in Cambodia with calcium intake below the RDA, as reported in the present study. Urban children had a higher intake of dairy products than their rural counterparts, which is similar to the results of the Chinese survey conducted among children aged 4–17 years. This result indicates that community-specific strategies must be developed and implemented to improve the quality of children’s diets [25]. In this study, the high percentage of children with nutrient intakes below the RDA might be because the nutrient intake was compared with the crude RDA values from a single 24-h-recall survey. The raw data almost overestimated the proportion of the population below a given cut-off irrespective of whether this is an estimated average requirement (EAR) or RDA [26,27].

### 4.3. Strengths and Limitations

The strength of the study is the availability of anthropometric and nutrient intake data of 2020 children from the whole geographical region of Cambodia, and children were representative of the total population aged 6–17 years (by area of residence and sex) in the country, based on weight factor adjustment. A limitation of this study is that the results of nutrient intakes in the present study were not adjusted to obtain the usual intake distribution, because only single-day intakes were collected by 24-h-recall and there is no available data for removing the day-to-day variability in intakes (within-person variation) in Cambodia. Additionally, the result did not include vitamin D data because no calculation system was used. The SEANUTS reported that vitamin D deficiency is widespread in all four countries, irrespective of sex and location. Vitamin D is an essential nutrient as it enhances calcium absorption for optimal bone health [13]. Vitamin D data should be obtained in future surveys to provide more comprehensive data. Furthermore, data from children less than 6 years old is needed to provide sufficient information about the nutritional status of Cambodian children, as shown in the SEANUTS.

## 5. Conclusions

In Cambodia, the overall prevalence of stunting was high and that of overweight and obesity was not as high compared with the SEANUTS. Among school-aged children in Cambodia, those living in rural areas had a higher percentage of undernutrition, while those living in urban areas had a higher percentage of overnutrition, which is similar to the results reported in the SEANUTS. In addition, the difference between the energy ratio of macronutrients and intake of food groups was based on the area of residence. Strategies for improving the nutritional status of school-aged children in Cambodia should not only focus on managing undernutrition, but should also emphasize developing approaches to determine the differences between the areas of residence. In addition, policies must be developed, planning must be conducted, and nutrition interventions must be applied among school-aged children in Cambodia.

## Figures and Tables

**Table 1:** Number of children who participated in the study by age group, sex, and area of residence.

Age Group (Years Old)	Boys		Girls		Total
	Rural	Urban	Rural	Urban	
6–9	182/482,826	50/105,367	207/475,028	55/106,799	494/1,170,020
10–12	203/392,246	48/84,140	227/372,058	54/74,235	532/922.679
13–15	260/386,764	50/94,126	291/368,983	69/80,156	670/930,029
16–17	128/239,170	38/57,473	120/236,176	38/53,970	324/586,789
Total	773/1,501,006	186/341,106	845/1,452,245	216/315,160	2020/3,609,517

Number of samples/estimated population. Estimated population size based on the census data from the 2013 Cambodia Inter-censal Population Survey.

**Table 2:** Anthropometric characteristics (mean values ± standard errors) ^†^ and prevalence (%) ^‡^ of under and overnutrition by age group and area of residence.

	**6–9 Years Old**				**10–12 Years Old**			
	**Boys**		**Girls**		**Boys**		**Girls**	
	**Rural**	**Urban**	**Rural**	**Urban**	**Rural**	**Urban**	**Rural**	**Urban**
H (cm) ^†^	118.3 ± 0.2	122.3 ± 0.4 ^b^	117.3 ± 0.2	121.7 ± 0.5 ^b^	131.8 ± 0.5	133.6 ± 1.0	134.3 ± 0.5	138.8 ± 1.1 ^b^
W (kg) ^†^	20.5 ± 0.1	22.9 ± 0.2 ^b^	19.8 ± 0.1	22.1 ± 0.3 ^b^	26.6 ± 0.3	29.0 ± 0.7 ^b^	28.5 ± 0.4	31.9 ± 0.9 ^b^
HAZ ^†^	−1.47 ± 0.04	−0.86 ± 0.06 ^b^	−1.48 ± 0.04	−0.88 ± 0.08 ^b^	−1.88 ± 0.07	−1.73 ± 0.14	−1.80 ± 0.07	−1.25 ± 0.15 ^b^
BAZ ^†^	−0.96 ± 0.04	−0.65 ± 0.07 ^b^	−0.97 ± 0.04	−0.72 ± 0.07 ^b^	−1.25 ± 0.07	−0.87 ± 0.14 ^a^	−1.13 ± 0.07	−0.80 ± 0.14 ^a^
Stunting ^‡^	27.5	14.0	29.0	10.9 ^b^	45.8	35.4	45.4	29.6 ^a^
Thinness ^‡^	12.1	4.0 ^a^	7.2	9.1	17.2	10.4	22.9	14.8
Over-w ^‡^	1.6	8.0 ^a^	1.0	1.8	1.0	4.2	3.5	5.6
Obesity ^‡^	0.5	4.0 ^a^	0.5	5.5	0.5	4.2 ^a^	0.0	0.0
	**13–15 Years Old**				**16–17 Years Old**			
	**Boys**		**Girls**		**Boys**		**Girls**	
	**Rural**	**Urban**	**Rural**	**Urban**	**Rural**	**Urban**	**Rural**	**Urban**
H (cm) ^†^	148.8 ± 0.6	154.1 ± 1.3 ^b^	148.7 ± 0.4	151.0 ± 0.8 ^a^	162.6 ± 0.6	166.8 ± 1.1 ^b^	153.9 ± 0.5	155.8 ± 0.9
W (kg) ^†^	37.9 ± 0.5	42.8 ± 1.2 ^b^	40.0 ± 0.4	42.3 ± 0.9 ^a^	50.1 ± 0.8	53.1 ± 1.4	45.9 ± 0.5	48.2 ± 0.9 ^a^
HAZ ^†^	−2.00 ± 0.07	−1.46 ± 0.16 ^b^	−1.63 ± 0.06	−1.33 ± 0.12 ^a^	−1.56 ± 0.08	−1.03 ± 0.14 ^b^	−1.32 ± 0.08	−1.06 ± 0.14
BAZ ^†^	−1.32 ± 0.06	−0.98 ± 0.14 ^a^	−0.88 ± 0.06	−0.66 ± 0.13	−1.04 ± 0.09	−1.03 ± 0.16	−0.66 ± 0.07	−0.48 ± 0.13
Stunting ^‡^	50.8	26.0 ^b^	30.9	17.4 ^a^	28.9	10.5 ^a^	20.0	18.4
Thinness ^‡^	26.5	14.0	16.2	10.1	11.7	13.2	4.2	7.9
Over-w ^‡^	0.8	2.0	4.5	5.8	2.3	5.3	0.8	0.0
Obesity ^‡^	0.0	2.0 ^a^	0.0	0.0	0.0	2.6	0.0	0.0
	**Boys**		**Girls**		**Total**		**All**	
	**Rural**	**Urban**	**Rural**	**Urban**	**Rural**	**Urban**		
H (cm) ^†^	139.3 ± 0.3	143.3 ± 0.6 ^b^	138.0 ± 0.2	140.9 ± 0.5 ^b^	138.7 ± 0.2	142.1 ± 0.4 ^b^	140.4 ± 0.2	
W (kg) ^†^	32.8 ± 0.3	36.3 ± 0.5 ^b^	32.9 ± 0.2	35.3 ± 0.4 ^b^	32.8 ± 0.2	35.8 ± 0.3 ^b^	34.3 ± 0.2	
HAZ ^†^	−1.77 ± 0.04	−1.28 ± 0.08 ^b^	−1.60 ± 0.03	−1.14 ± 0.07 ^b^	−1.68 ± 0.03	−1.21 ± 0.05 ^b^	−1.45 ± 0.03	
BAZ ^†^	−1.17 ± 0.04	−0.88 ± 0.07 ^b^	−0.94 ± 0.03	−0.68 ± 0.07 ^b^	−1.06 ± 0.03	−0.77 ± 0.05 ^b^	−0.92 ± 0.03	
Stunting ^‡^	40.4	22.0 ^b^	32.8	19.0 ^b^	36.4	20.4 ^b^	33.2	
Thinness ^‡^	18.2	10.2 ^b^	14.1	10.6	16.1	10.4 ^b^	15.0	
Over-w ^‡^	1.3	4.8 ^b^	2.8	3.7	2.1	4.2 ^a^	2.5	
Obesity ^‡^	0.3	3.2 ^b^	0.1	1.4 ^a^	0.2	2.2 ^b^	0.6	

^†^, Data were presented as mean values ± standard errors. Mean values were based on analysis of covariance after adjusting for age. ^‡^, % difference between the urban and rural children for each sex based on the χ^2^ test. H, Height; W, Weight; Over-w, Overweight; HAZ, Height for age; BAZ, Body mass index for age; stunting was defined as HAZ <−2SD. Thinness was defined as BAZ <−2SD. Overweight and obese were defined as BA >1SD to ≤2SD and >2SD. ^a^, *p*-value < 0.05, ^b^, *p*-value < 0.01.

**Table 3:** Intake of energy and selected nutrients and the percentage (%) from total energy by age group and area of residence (mean values ± standard errors) ^†^.

**Energy & Nutrients**	**6–9 Years Old**				**10–12 Years Old**			
	**Boys**		**Girls**		**Boys**		**Girls**	
	**Rural**	**Urban**	**Rural**	**Urban**	**Rural**	**Urban**	**Rural**	**Urban**
E (kcal)	1408 ± 18	1438 ± 31	1281 ± 19	1382 ± 37 ^a^	1571 ± 39	1630 ± 80	1460 ± 37	1608 ± 75
Pro (g)	39.0 ± 0.6	41.2 ± 1.0	34.6 ± 0.6	39.9 ± 1.2 ^b^	42.7 ± 1.5	47.6 ± 3.0	40.2 ± 1.2	44.3 ± 2.5
Fat (g)	23.7 ± 0.6	30.7 ± 1.0 ^b^	21.8 ± 0.6	30.3 ± 1.1 ^b^	23.8 ± 1.0	27.4 ± 2.0	22.4 ± 0.9	31.1 ± 1.9 ^b^
CHO (g)	261.4 ± 3.4	252.3 ± 5.7	237.7 ± 3.7	240.3 ± 7.1	297.5 ± 7.3	300.9 ± 15.1	275.6 ± 7.1	287.5 ± 14.6
VA (μg)	277 ± 11	294 ± 20	246 ± 12	275 ± 23	285 ± 20	418 ± 41 ^b^	327 ± 25	346 ± 50
Fe (mg)	7.7 ± 0.1	7.7 ± 0.2	6.4 ± 0.2	8.1 ± 0.3 ^b^	8.9 ± 0.3	9.3 ± 0.7	8.3 ± 0.3	8.9 ± 0.6
Ca (mg)	330 ± 8	361 ± 13 ^a^	311 ± 9	358 ± 17 ^a^	342 ± 16	428 ± 32 ^a^	411 ± 16	390 ± 34
Pro (E%) ^‡^	11.3 ± 0.1	11.4 ± 0.2	10.8 ± 0.1	11.6 ± 0.2 ^b^	10.8 ± 0.2	11.6 ± 0.4	11.0 ± 0.2	11.1 ± 0.4
Fat (E%) ^‡^	14.9 ± 0.2	18.7 ± 0.4 ^b^	15.1 ± 0.3	19.3 ± 0.5 ^b^	13.4 ± 0.4	15.2 ± 0.8 ^a^	13.6 ± 0.4	17.4 ± 0.8 ^b^
CHO (E%) ^‡^	73.8 ± 0.3	69.9 ± 0.5 ^b^	74.0 ± 0.3	69.1 ± 0.6 ^b^	75.8 ± 0.5	73.3 ± 1.0 ^a^	75.4 ± 0.5	71.5 ± 1.0 ^b^
**Energy & Nutrients**	**13–15 Years Old**				**16–17 Years Old**			
	**Boys**		**Girls**		**Boys**		**Girls**	
	**Rural**	**Urban**	**Rural**	**Urban**	**Rural**	**Urban**	**Rural**	**Urban**
E (kcal)	1851 ± 38	1802 ± 86	1584 ± 34	1629 ± 71	2023 ± 52	2143 ± 95	1668 ± 51	1555 ± 93
Pro (g)	51.6 ± 1.3	53.0 ± 2.9	44.0 ± 1.1	49.0 ± 2.2 ^a^	54.7 ± 1.7	65.5 ± 3.1 ^b^	47.6 ± 1.9	50.2 ± 3.4
Fat (g)	28.4 ± 1.1	33.6 ± 2.4	26.7 ± 0.9	32.3 ± 0.2 ^b^	32.3 ± 1.7	39.0 ± 3.0	31.1 ± 1.7	36.1 ± 3.0
CHO (g)	347 ± 7	323.1 ± 16	291.6 ± 6.6	285.5 ± 13.5	375.9 ± 9.8	381.0 ± 18.0	298.5 ± 8.9	257.5 ± 16.1 ^a^
VA (μg)	423 ± 22	418 ± 50	483 ± 68	386 ± 141	503 ± 44	569 ± 81	431 ± 31	397 ± 56
Fe (mg)	10.2 ± 0.3	9.9 ± 0.7	9.2 ± 0.3	9.9 ± 0.6	11.2 ± 0.4	12.2 ± 0.8	10.1 ± 0.5	9.5 ± 0.9
Ca (mg)	452 ± 17	424 ± 39	424 ± 14	396 ± 30	447 ± 22	501 ± 40	415 ± 23	472 ± 42
Pro (E%) ^‡^	11.2 ± 0.2	11.9 ± 0.4	11.2 ± 0.1	12 ± 0.3 ^a^	11.0 ± 0.2	12.2 ± 0.4 ^b^	11.4 ± 0.2	12.8 ± 0.4 ^b^
Fat (E%) ^‡^	13.4 ± 0.4	16.8 ± 0.8 ^b^	15.1 ± 0.4	17.5 ± 0.8 ^b^	14.1 ± 0.6	16.3 ± 1.0	16.3 ± 0.6	20.8 ± 1.1 ^b^
CHO (E%) ^‡^	75.4 ± 0.4	71.3 ± 1.0 ^b^	73.7 ± 0.4	70.5 ± 0.9 ^b^	75.0 ± 0.6	71.5 ± 1.2 ^a^	72.3 ± 0.7	66.4 ± 1.2 ^b^
**Energy & Nutrients**	**Boys**		**Girls**		**Total**		**All**	
	**Rural**	**Urban**	**Rural**	**Urban**	**Rural**	**Urban**		
E (kcal)	1700 ± 20	1734 ± 41	1489.2 ± 19	1544 ± 37	1595 ± 14	1638 ± 28	1616 ± 16	
Pro (g)	46.8 ± 0.7	51.1 ± 1.4 ^b^	41.2 ± 0.6	45.6 ± 1.2 ^b^	44.0 ± 0.5	48.3 ± 0.9 ^b^	46.2 ± 0.5	
Fat (g)	26.7 ± 0.6	32.5 ± 1.2 ^b^	25.0 ± 0.5	32.1 ± 1.0 ^b^	25.8 ± 0.4	32.3 ± 0.8 ^b^	29.1 ± 0.4	
CHO (g)	318.4 ± 3.8	311.0 ± 7.8	275.2 ± 3.6	269.1 ± 7.1	296.9 ± 2.6	289.7 ± 5.3	293.3 ± 2.9	
VA (μg)	365 ± 13	419 ± 26	377 ± 25	346 ± 50	371 ± 15	383 ± 29	377 ± 16	
Fe (mg)	9.4 ± 0.2	9.7 ± 0.3	8.4 ± 0.2	9.1 ± 0.3	8.9 ± 0.1	9.4 ± 0.2	9.1 ± 6.8	
Ca (mg)	394 ± 9	426 ± 18	391 ± 8	399 ± 17	393 ± 6	412 ± 12	402 ± 0	
Pro (E%) ^‡^	11.1 ± 0.1	11.7 ± 0.2 ^b^	11.1 ± 0.1	11.8 ± 0.2 ^b^	11.1 ± 0.1	11.8 ± 0.1 ^b^	11.4 ± 0.1	
Fat (E%) ^‡^	13.8 ± 0.2	16.8 ± 0.4 ^b^	14.9 ± 0.2	18.5 ± 0.4 ^b^	14.4 ± 0.2	17.7 ± 0.3 ^b^	16.0 ± 0.2	
CHO (E%) ^‡^	75.1 ± 0.3	71.5 ± 0.5 ^b^	74.0 ± 0.3	69.7 ± 0.5 ^b^	74.5 ± 0.2	70.6 ± 0.4 ^b^	72.6 ± 0.2	

^†^, Data were presented as mean values ± standard errors. Mean values were based on analysis of covariance after adjusting for age. ^a^, *p*-value < 0.05, ^b^, *p*-value < 0.01. ^‡^, E%, percentage from total energy, E, Energy; Pro, Protein; CHO, Carbohydrate; VA, Vitamin A; Fe, Iron; Ca, Calcium.

**Table 4:** Percentage ^†^ of children consuming selected energy, macronutrients, and micronutrients below the Cambodian Recommended Dietary Allowance (CAM-RDA) by age group, sex, and area of residence.

**Energy and Nutrients**	**6–9 Years Old**				**10–12 Years Old**			
	**Boys**		**Girls**		**Boys**		**Girls**	
	**Rural**	**Urban**	**Rural**	**Urban**	**Rural**	**Urban**	**Rural**	**Urban**
Energy	58.8	56.0	72.0	65.5	71.9	70.8	73.6	61.1
Protein	28.0	20.0	46.9	30.9 ^a^	53.7	50.0	59.0	46.3
Vitamin A	83.5	80.0	85.5	87.3	87.7	79.2	85.0	81.5
Calcium	84.1	76.0	83.6	80.0	95.1	89.6	89.0	92.6
Iron	33.5	24.0	44.9	30.9	18.2	14.6	70.5	64.8
**Energy and Nutrients**	**13–15 Years Old**				**16–17 Years Old**			
	**Boys**		**Girls**		**Boys**		**Girls**	
	**Rural**	**Urban**	**Rural**	**Urban**	**Rural**	**Urban**	**Rural**	**Urban**
Energy	74.2	78.0	78.4	76.8	78.1	76.3	84.2	89.5
Protein	41.2	40.0	46.4	33.3 ^a^	34.3	21.1	40.0	31.6
Vitamin A	73.5	80.0	78.4	81.2	73.4	76.3	80.0	73.7
Calcium	89.2	94.0	82.1	85.5	88.3	76.3	82.5	68.4
Iron	48.5	54.0	65.6	59.4	22.7	10.5	40.8	44.7
**Energy and** **Nutrients**	**Boys**		**Girls**		**Total**		**All**	
	**Rural**	**Urban**	**Rural**	**Urban**	**Rural**	**Urban**		
Energy	70.6	69.9	76.3	72.2	73.6	71.1	73.1	
Protein	40.2	33.3	49.0	35.6 ^b^	44.8	34.6 ^b^	42.8	
Vitamin A	79.6	79.0	82.1	81.5	80.9	80.3	80.8	
Calcium	89.4	84.4	84.4	82.9	86.8	83.6	86.1	
Iron	32.7	26.9	58.3	50.9	46.1	39.8 ^a^	44.9	

^†^, % difference between the urban and rural children of each sex based on the χ^2^ test. ^a^, *p*-value < 0.05, ^b^, *p*-value < 0.01.

**Table 5:** Intake of food groups by age group and the area of residence (mean values ± standard errors) ^†^.

**Food Group (g)**	**6–9 Years Old**				**10–12 Years Old**			
	**Boys**		**Girls**		**Boys**		**Girls**	
	**Rural**	**Urban**	**Rural**	**Urban**	**Rural**	**Urban**	**Rural**	**Urban**
Staple foods ^1^	583.1 ± 9.0	569.2 ± 15.3	520.3 ± 9.3	465.9 ± 18.1 ^b^	693.0 ± 18.7	675.5 ± 38.5	625.0 ± 17.4	616.9 ± 35.8
Legume, nuts	18.0 ± 2.9	47.0 ± 4.9 ^b^	21.6 ± 2.5	25.6 ± 4.9	18.8 ± 4.3	35.4 ± 8.7	25.4 ± 5.7	24.3 ± 11.7
Veges ^2^, fruits	207.4 ± 7.6	143.4 ± 13.0 ^b^	196.1 ± 8.8	196.0 ± 17.1	207.1 ± 11.7	199.2 ± 24.0	225.5 ± 13.3	230.4 ± 27.2
Meats, eggs	50.3 ± 2.1	73.3 ± 3.6 ^b^	38.1 ± 1.8	71.6 ± 3.6 ^b^	48.3 ± 3.9	73.2 ± 8.0 ^b^	42.7 ± 3.3	73.4 ± 6.7 ^b^
Fish, shellfish	43.9 ± 1.6	29.0 ± 2.7 ^b^	41.3 ± 1.7	28.0 ± 3.3 ^b^	42.6 ± 3.8	46.1 ± 7.9	47.8 ± 3.1	41.5 ± 6.3
Dairy foods ^3^	13.8 ± 2.1	37.6 ± 3.5 ^b^	15.8 ± 3.1	44.2 ± 6.0 ^b^	10.8 ± 2.7	23.7 ± 5.5 ^a^	15.4 ± 3.3	30.6 ± 6.8 ^a^
Fats, oils	5.0 ± 0.2	5.6 ± 0.3	4.5 ± 0.2	5.4 ± 0.4 ^a^	4.7 ± 0.3	5.8 ± 0.7	3.4 ± 0.3	8.2 ± 0.6 ^b^
Sugars	10.0 ± 0.4	8.7 ± 0.6	13.5 ± 0.8	15.0 ± 1.6	13.1 ± 1.1	13.7 ± 2.2	11.6 ± 1.0	17.0 ± 2.1 ^a^
Confectionary ^4^	34.9 ± 1.9	33.9 ± 3.2	36.8 ± 1.9	39.4 ± 3.8	35.2 ± 4.0	20.9 ± 8.3	32.1 ± 3.2	27.6 ± 6.6
Condiments ^5^	14.6 ± 0.5	14.5 ± 0.8	13.9 ± 0.6	17.5 ± 1.2 ^b^	15.6 ± 1.2	25.4 ± 2.4 ^b^	15.9 ± 0.9	18.9 ± 1.9
Beverages ^6^	45.9 ± 4.4	93.3 ± 7.5 ^b^	42.8 ± 3.8	77.6 ± 7.5 ^b^	42.1 ± 6.8	84.9 ± 13.9 ^b^	70.3 ± 9.1	102.2 ± 18.7
**Food Group (g)**	**13–15 Years Old**				**16–17 Years Old**			
	**Boys**		**Girls**		**Boys**		**Girls**	
	**Rural**	**Urban**	**Rural**	**Urban**	**Rural**	**Urban**	**Rural**	**Urban**
Staple foods ^1^	814.0 ± 18.0	754.1 ± 41.2	634.0 ± 15.4	577.5 ± 31.6	908.5 ± 26.1	875.5 ± 47.9	614.0 ± 20.9	525.9 ± 37.7 ^a^
Legume, nuts	39.8 ± 6.5	18.9 ± 14.7	34.2 ± 5.7	66.0 ± 11.7 ^a^	44.3 ± 8.2	36.8 ± 15.1	29.7 ± 5.7	26.2 ± 10.3
Veges ^2^, fruits	257.5 ± 14.4	258.7 ± 32.9	278.0 ± 15.3	310.7 ± 31.6	286.4 ± 20.9	310.5 ± 38.3	305.7 ± 22.4	266.1 ± 40.3
Meats, eggs	60.7 ± 3.9	85.1 ± 9.0 ^a^	58.0 ± 3.7	82.3 ± 7.5 ^b^	74.6 ± 6.5	111.1 ± 11.8 ^b^	69.0 ± 5.7	96.0 ± 10.3 ^a^
Fish, shellfish	55.7 ± 3.6	61.8 ± 8.3	48.9 ± 3.0	55.3 ± 6.1	53.8 ± 5.2	70.4 ± 9.5	49.7 ± 5.0	56.2 ± 8.9
Dairy foods ^3^	16.2 ± 3.1	23.3 ± 7.1	16.2 ± 3.2	28.9 ± 6.5	6.2 ± 2.7	15.2 ± 4.9	18.4 ± 4.5	44.7 ± 8.0 ^b^
Fats, oils	5.7 ± 0.4	8.0 ± 0.9 ^a^	5.1 ± 0.3	8.0 ± 0.7 ^b^	7.4 ± 0.7	9.5 ± 1.2	6.6 ± 0.7	8.4 ± 1.2
Sugars	17.5 ± 1.3	14.3 ± 2.9	15.6 ± 1.0	16.6 ± 2.0	16.5 ± 1.3	15.0 ± 2.4	17.6 ± 1.7	12.8 ± 3.1
Confectionary ^4^	30.9 ± 3.4	28.7 ± 7.8	29.3 ± 2.7	19.9 ± 5.6	19.5 ± 4.0	28.8 ± 7.4	36.4 ± 5.4	12.1 ± 9.8 ^a^
Condiments ^5^	21.1 ± 1.1	21.9 ± 2.6	22.4 ± 1.3	23.8 ± 2.8	24.8 ± 1.7	23.4 ± 3.2	23.2 ± 2.1	34.0 ± 3.8 ^a^
Beverages ^6^	83.3 ± 9.0	124.8 ± 20.6	75.6 ± 8.1	79.9 ± 16.6	119.9 ± 18.7	198.4 ± 34.3 ^a^	99.8 ± 16.9	151.7 ± 30.4
**Food Group (g)**	**Boys**		**Girls**		**Total**		**All**	
	**Rural**	**Urban**	**Rural**	**Urban**	**Rural**	**Urban**		
Staple foods ^1^	673.5 ± 7.2	648.9 ± 13.3	547.6 ± 6.9	520.5 ± 13.6 ^b^	672.4 ± 6.6	628.3 ± 13.3 ^b^	650.3 ± 7.4	
Legume, nuts	24.8 ± 2.3	41.4 ± 4.2 ^b^	25.9 ± 2.1	34.0 ± 4.2	29.0 ± 2.1	36.2 ± 4.2	32.6 ± 2.3	
Veges ^2^, fruits	223.4 ± 5.8	186.6 ± 10.7 ^b^	230.7 ± 6.4	234.5 ± 12.7	242.3 ± 5.6	239.0 ± 11.2	240.6 ± 6.2	
Meats, eggs	54.1 ± 1.6	79.6 ± 3.0 ^b^	46.4 ± 1.5	77.2 ± 2.9 ^b^	53.9 ± 1.5	82.2 ± 2.9 ^b^	68.0 ± 1.6	
Fish, shellfish	46.8 ± 1.4	40.1 ± 2.5 ^a^	45.0 ± 1.3	39.4 ± 2.6	48.0 ± 1.3	47.9 ± 2.6	48.0 ± 1.4	
Dairy foods ^3^	13.1 ± 1.4	31.8 ± 2.6 ^b^	16.0 ± 1.8	38.9 ± 3.6 ^b^	14.3 ± 1.3	30.9 ± 2.6 ^b^	22.6 ± 1.5	
Fats, oils	5.3 ± 0.2	6.4 ± 0.3 ^b^	4.6 ± 0.1	6.8 ± 0.3 ^b^	5.1 ± 0.1	7.3 ± 0.3 ^b^	6.2 ± 0.2	
Sugars	12.5 ± 0.4	10.9 ± 0.7	14.0 ± 0.5	15.5 ± 1.0	14.3 ± 0.4	14.2 ± 0.8	14.3 ± 0.5	
Confectionary ^4^	32.9 ± 1.4	30.3 ± 2.6	34.3 ± 1.4	30.0 ± 2.7	32.1 ± 1.3	26.6 ± 2.6	29.3 ± 1.4	
Condiments ^5^	16.9 ± 0.4	18.2 ± 0.8	17.1 ± 0.5	20.9 ± 1.0 ^b^	18.7 ± 0.5	22.0 ± 0.9 ^b^	20.3 ± 0.5	
Beverages ^6^	59.3 ± 3.7	108.8 ± 6.8 ^b^	60.8 ± 3.5	90.7 ± 6.9 ^b^	69.6 ± 3.5	109.6 ± 7.0 ^b^	89.6 ± 3.9	

^†^, Data were presented as mean values ± standard errors. Mean values were based on analysis of covariance after adjusting for age. ^a^, *p*-value < 0.05, ^b^, *p*-value < 0.01. ^1^, Staple foods included cereals, bread, noodle, tuber, and starchy food; ^2^, Veges, Vegetables; ^3^, Dairy foods included milk and milk products; ^4^, Confectionary included snacks and sweets; ^5^, Condiments included sauces and spices; ^6^, Beverages included juice, energy drink, tea, and coffee.
